# Deep venous thrombosis after office vasectomy: a case report

**DOI:** 10.1186/1752-1947-4-242

**Published:** 2010-08-04

**Authors:** David A Cooke, Philip Zazove

**Affiliations:** 1 Department of Internal Medicine, University of Michigan Medical Center, Ann Arbor, Michigan, USA; 2 Department of Family Medicine, University of Michigan Medical Center, Ann Arbor, Michigan, USA

## Abstract

**Introduction:**

Postoperative pulmonary embolism is considered a complication of major surgery. However, thromboembolism can also occur following minor procedures. We report a case of a major embolic event following a straightforward office vasectomy.

**Case presentation:**

A healthy 35-year-old Asian man underwent an uncomplicated office vasectomy. Soon after, he noticed vague chest pain and dyspnea. Lower extremity Doppler ultrasound revealed acute venous thrombosis. A computer-assisted tomography angiogram revealed extensive bilateral pulmonary emboli. Extensive laboratory work-up failed to identify thrombophilia. He has not had any recurrences in the eight years since the initial presentation.

**Conclusion:**

This case highlights that major embolic events can follow minor office procedures. Patients with suggestive findings should be investigated aggressively.

## Introduction

Pulmonary embolism is a well-known complication of major surgery but it is not always appreciated that it can occur even after minor interventions. Thromboembolism has been reported after outpatient surgeries of many types. However, there are very few reports of thromboembolism associated with an office vasectomy. We believe that, although the incidence of this complication is low, it does occur and physicians should be aware of this if a patient presents with symptoms suggestive of an embolic event.

## Case presentation

A 35-year-old Asian man without a significant medical history presented to our health center for elective outpatient vasectomy. The procedure was performed bilaterally using the no-scalpel approach in an office setting over 30 minutes without any apparent incident or complication and he was discharged. He returned home and reported that he slept for about two hours in bed. Upon arising, he noticed that he felt somewhat short of breath and experienced dyspnea on exertion as well as vague substernal chest pain; none of his symptoms were sufficiently severe to lead him to seek immediate medical attention.

He subsequently presented to his primary care physician (PCP) four days following the vasectomy procedure because of continued feelings of shortness of breath and chest pain. The rest of the history was unremarkable, as was his physical examination and electrocardiogram. He had no swelling, tenderness, warmth or redness of his legs. There was some mild tenderness and ecchymoses at the operative site but no edema or swelling.

**Figure 1:**
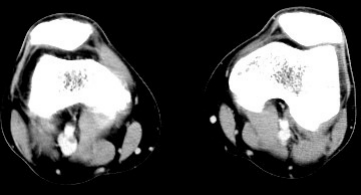


**Figure 2:**
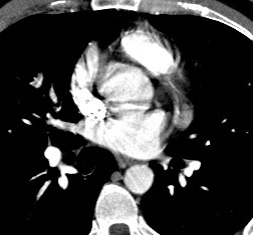


**Figure 3:**
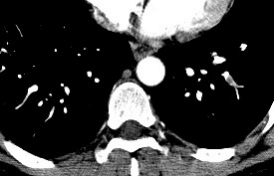


In light of his recent procedure and his symptoms, lower extremity Doppler ultrasound studies were ordered and performed 36 hours after his visit to the PCP. The Doppler studies demonstrated venous thrombosis in the right popliteal vein. A computed tomogram (CT) angiogram was immediately arranged which re-demonstrated a clot in the right popliteal vein (Figure 1). Additionally, it also demonstrated large, multiple, bilateral pulmonary emboli (Figures 2 and 3).

He did not have any prior history of deep venous thrombosis (DVT) and was not aware of any family history of the disorder. He was taking no medications at the time of the vasectomy. He was a non-smoker. An extensive laboratory work-up was performed in search of any underlying disorders predisposing to thromboembolism (Table 1). All studies returned within the normal limits. D-Dimer and Factor VIII levels were not checked, as these tests were not routinely utilized as part of a thromboembolic workup at the time of the event.

**Table 1:** Selected test values for patient.

Test	Patient result	Lab normal
White blood count	5.8 K/mm^3^	4.0 - 10.0 K/mm^3^

Red blood count	5.13 K/mm^3^	4.50 - 5.90 K/mm^3^

Hemoglobin	15.3 g/dL	13.0 - 17.3 g/dL

Platelet count	206 K/mm^3^	150 - 450 K/mm^3^

Westergren sedimentation rate	8 mm/h	0 - 15 mm/h

Prothrombin time	13.2 s	10.5 - 13.5 s

International Normalized Ratio	1.0	

Partial thromboplastin time	31.3 s	25.0 - 32.6 s

Dilute Russell viper venom time	25.8 s	24.8 - 38.0 s

TT inhibition	0.9 (1:100) 0.8 (1:1000)	0.0 - 1.2 0.0 - 1.2

Homocysteine	12 μmol/L	5 - 15 μmol/L

Protein C activity	115%	81% - 160%

Protein C antigen	92%	60% - 106%

Protein S antigen, free	50%	43% - 132%

Antinuclear antibody	1:80 (speckled pattern)	Negative

lgG Phospholipid antibody	11 GPL	0 - 22 GPL

lgM phospholipid antibody	5 MPL	0 - 10 MPL

Antithrombin III activity	101%	82% - 119%

Antithrombin III antigen	32.0 mg/dL	20.0 - 32.0 mg/dL

Factor V Leiden mutation	Negative	Negative

The patient was treated initially for his pulmonary embolism as an outpatient with enoxaparin and then switched to warfarin for six months. The patient tolerated this therapy well and did not develop any bleeding complications or symptoms suggestive of recurrent embolism. A CT angiography of the chest, pelvis and legs was repeated 76 days after the initial study to confirm resolution of the thrombi. This demonstrated complete resolution of the pulmonary emboli, with no residual clot in the pulmonary or lower extremity venous systems. Symptomatically, he has also returned to baseline by this point. To date, he has not had any evidence of recurrent thrombosis or thromboembolism.

## Discussion

We present here the case of a previously healthy man who developed extensive pulmonary emboli shortly after an elective vasectomy procedure. We believe there is a direct relationship between the two events, most likely mediated by venous stasis and inflammation from procedural trauma.

It is impossible to exclude a chance association in our case between the thromboembolic event and the vasectomy. However, our patient had no identifiable underlying hypercoaguable state and he has not had any recurrent thromboembolism in the eight years since his vasectomy. These factors strongly suggest that his thromboembolism resulted from the procedure.

While the association we propose is not generally known, there is limited precedent in the medical literature. Two articles by Roberts [1,2] in 1968 and 1971 hypothesized there is an association between vasectomy and thrombophlebitis. However, these cases reported a delay of several years between the vasectomies and the presumed related thrombotic events. Another case report in 1973 posits a relationship but, again, several months elapsed between vasectomy and the thrombotic event [3]. Recently, Teachey [4] reported a case of pulmonary embolism occurring soon after vasectomy and we believe the similarity of this case adds support to our argument.

The operating physician in this case has performed hundreds of vasectomies over a period of more than 20 years and this is the first such a complication that he has seen. Together with the very small number of similar reports, this suggests that post-vasectomy thromboembolism represents an extremely rare complication of the procedure.

It is interesting that this occurred despite using the no-scalpel vasectomy technique, which is known to be quicker and less traumatic than the traditional approach. We wondered whether the fact that a resident was involved in doing the vasectomy on our patient was a factor in the development of postoperative complications. The attending surgeon usually completes most vasectomies in 15-20 min; in this case, the procedure probably lasted around 30 or even 35 min. The literature suggests that a resident performing a procedure, under the close supervision of a faculty (which was the case in this patient), does not increase the risk of complications. This has been studied using a variety of procedures, including cardiac, otolaryngologic and general surgery situations [5-10].

## Conclusion

This case highlights the need to be aware that major embolic events can occur after even minor office procedures. Patients at risk for developing clots should be managed appropriately before and during the surgery in order to reduce the chances of a problem. In addition, patients presenting with suggestive symptoms and signs of a DVT or pulmonary embolus after a procedure should be investigated aggressively.

## Abbreviations

CT: computed tomogram; DVT: deep venous thrombosis; PCP: primary care physician.

## Competing interests

The authors declare that they have no competing interests.

## Authors' contributions

PZ and DC both directly participated in the care of this patient, both contributed substantially to the text of the article and the literature review. Both authors read and approved the final manuscript.

## Consent

Written informed consent was obtained from the patient for publication of this case report and accompanying images. A copy of the written consent is available for review by the Editor-in-Chief of this journal.
